# Homocysteine in the Cardiovascular Setting: What to Know, What to Do, and What Not to Do

**DOI:** 10.3390/jcdd12100383

**Published:** 2025-09-27

**Authors:** Saverio D’Elia, Mariarosaria Morello, Gisella Titolo, Valentina Maria Caso, Achille Solimene, Ettore Luisi, Chiara Serpico, Andrea Morello, Lucia La Mura, Francesco S. Loffredo, Francesco Natale, Paolo Golino, Giovanni Cimmino

**Affiliations:** 1 Cardiology Unit, Azienda Ospedaliera Universitaria Luigi Vanvitelli, 80138 Naples, Italy; saveriodelia85@gmail.com; 2 Department of Advanced Medical and Surgical Sciences, University of Campania Luigi Vanvitelli, Piazza Luigi Miraglia, 2, 80138 Naples, Italy; gisellatitolo@gmail.com; 3 Department of Translational Medical Sciences, Section of Cardiology, University of Campania Luigi Vanvitelli, 80138 Naples, Italy; mariarosaria.morello@gmail.com (M.M.); achille.solimene@unicampania.it (A.S.); ettore.luisi@studenti.unicampania.it (E.L.); chiara.serpico@studenti.unicampania.it (C.S.); francesco.loffredo@unicampania.it (F.S.L.); paolo.golino@unicampania.it (P.G.); 4 Department of Precision Medicine, University of Campania Luigi Vanvitelli, 80131 Naples, Italy; valentinamaria.caso@unicampania.it; 5 Pharmacy Unit, Monaldi Hospital, 80131 Naples, Italy; 6 Laboratory Medicine Unit, Azienda Sanitaria Regionale Molise, Antonio Cardarelli Hospital, 86100 Campobasso, Italy; dr_andrea.morello@hotmail.it; 7 Department of Advanced Biomedical Sciences, Federico II University of Naples, 80138 Naples, Italy; lucia.lamura@unina.it; 8 Vanvitelli Cardiology and Intensive Care Unit, Monaldi Hospital, 80131 Naples, Italy; natalefrancesco@hotmail.com; 9 Department of Life Science, Health, and Health Professions, Link Campus University, 00165 Rome, Italy

**Keywords:** homocysteine, cardiovascular risk, folate, MTHFR, B vitamins, endothelial dysfunction, nutraceuticals, precision medicine

## Abstract

Homocysteine has long been studied as a potential cardiovascular risk factor due to its biochemical role in endothelial dysfunction, oxidative stress, inflammation, and thrombogenesis. Despite strong epidemiological and mechanistic support, the translation of homocysteine-lowering interventions into clinical benefit remains controversial. This non-systematic review aims to clarify the current understanding of homocysteine in the cardiovascular setting by distinguishing between well-established facts, clinically relevant interventions, and persistent misconceptions. We first revisit the historical emergence of homocysteine as a cardiovascular biomarker and explore its pathophysiological mechanisms, including endothelial damage, atherosclerosis progression, and prothrombotic effects—supported by in vitro and animal model studies. Subsequently, we evaluate evidence-based interventions such as B-vitamin supplementation (folate, B6, B12), lifestyle modifications, and the clinical relevance of homocysteine monitoring in specific populations (e.g., MTHFR mutations, chronic kidney disease). We then discuss common pitfalls, including the overinterpretation of genetic variants, the inappropriate use of supplementation, and the overreliance on surrogate biomarkers in clinical trials. Although elevated homocysteine remains a reproducible biomarker of cardiovascular risk, current evidence does not support routine intervention in unselected populations. A precision medicine approach—targeting high-risk subgroups and integrating homocysteine into broader cardiometabolic management—may help unlock its therapeutic relevance. Future pharmacological strategies should prioritize mechanistic insight, patient stratification, and clinically meaningful endpoints.

## 1. Introduction

Elevated plasma homocysteine—a sulfur-containing amino acid arising from methionine metabolism—has been proposed as an independent biomarker and potential therapeutic target for cardiovascular disease (CVD), due to its capacity to impair endothelial function, enhance oxidative stress, promote vascular smooth muscle proliferation, and increase thrombogenicity [1]. Meta-analyses of large-scale prospective cohort studies report a consistent dose–response relationship, indicating that a 5 µmol/L increase in homocysteine corresponds to a 20–30% higher risk of coronary artery disease and a 60% elevated stroke risk [2]. More recent evidence has reinforced this link: a 2022 systematic review and meta-analysis reported that every 5 µmol/L increase in plasma homocysteine was associated with an ≈22% higher risk of coronary heart disease [3], while a 2025 umbrella review confirmed robust associations of homocysteine with multiple cardiovascular outcomes, integrating both observational and Mendelian randomization data [4]. Genetic evidence, particularly Mendelian randomization studies involving the MTHFR C677T polymorphism—known to elevate homocysteine levels—initially appeared to support a causal link [5].

Recent meta-analyses have confirmed that this polymorphism is associated with an increased risk of myocardial infarction, particularly in young and middle-aged Caucasians.

In a pooled analysis of 8140 MI cases and 10,522 controls, individuals with the TT genotype had an OR of 1.183 (95% CI: 1.076–1.300) compared with CT carriers [6]. Other Mendelian randomization studies have clarified that genetically elevated homocysteine is not causally associated with congestive heart failure or cardiomyopathy. In a large two-sample MR including 218,792 CHF cases and 159,811 cardiomyopathy cases, no causal relationship was observed [7]. These findings suggest that homocysteine-lowering therapy may not influence the incidence or outcomes of these specific cardiovascular conditions.

However, interventional randomized controlled trials (RCTs) aimed at lowering homocysteine through supplementation with folic acid, vitamin B6, and B12—which achieve biochemical reductions of approximately 20–25%—have largely failed to demonstrate significant cardiovascular benefit, including myocardial infarction, coronary heart disease, or all-cause mortality [8,9]). Only modest reductions in stroke incidence (circa 10–12%) have been observed in subgroup analyses, though their clinical relevance remains debated [10].

The discordance between epidemiological/genetic correlations and null clinical outcomes raises critical questions: is homocysteine merely a passive indicator of vascular pathology, or could it represent a context-dependent pharmacological target? Moreover, heterogeneity in baseline folate status, homocysteine levels, genetic background (e.g., MTHFR genotype), dosage regimens, and follow-up duration across trials may obscure subpopulation-specific effects [11]. Further complicating interpretation are theoretical concerns about high-dose folic acid supplementation, including possible epigenetic modulation or the unanticipated promotion of neoplastic processes, which—although unproven—warrant careful pharmacovigilance [12].

Given these complexities, it is crucial for pharmacological research to differentiate well-supported findings from conflicting or overstated claims, to investigate potential therapeutic niches (e.g., patient subsets with MTHFR variants or low baseline folate), and to design precision-targeted RCTs that integrate mechanistic biomarkers alongside clinical endpoints.

A rigorous, evidence-based reassessment of homocysteine’s modulatory potential may reveal whether targeted pharmacotherapy can yield meaningful cardiovascular benefits where broad supplementation has failed.

## 2. What to Know: Molecular Mechanism and Biochemical Pathways

### 2.1. Historical Background

The recognition of homocysteine’s role in vascular pathology dates back to 1962, when Carson and Neill described homocystinuria—a rare inherited disorder characterized by extremely elevated homocysteine levels and premature vascular complications, including arterial thrombosis in children [13]. This seminal discovery established a crucial link between sulfur-amino acid metabolism and vascular disease.

In 1969, Kilmer McCully expanded upon this concept with pioneering histopathological studies of autopsied homocystinuric children, documenting widespread arterial atherosclerosis and proposing that hyperhomocysteinemia itself—independent of classic lipid pathways—could induce atherothrombotic vascular injury [14]. Animal experiments followed, demonstrating that the administration of homocysteine or its thiolactone forms to rabbits and monkeys produced aortic lesions and thromboses similar to human atherosclerosis, thereby reinforcing its pathogenic potential [15].

Prospective epidemiological research in the 1990s further substantiated homocysteine as a cardiovascular risk marker: a meta-analysis by Boushey and colleagues in 1995 demonstrated a robust, independent association between plasma homocysteine and coronary artery disease (adjusted RR ~1.5 per 5 µmol/L increase). This phase also saw the discovery of MTHFR C677T polymorphism by Frosst et al. [16], which highlighted a genetic determinant of mild hyperhomocysteinemia, particularly in low-folate settings, and cemented the hypothesis of a genetic–biochemical disease axis. 

Mendelian randomization studies in the early 2000s explored this genetic link further. Notably, Klerk et al. [5] analyzed the MTHFR variant in over 11,000 subjects, reporting a significant increase in coronary heart disease risk among homozygotes, reinforcing the hypothesis of a potential causal role for homocysteine 

The following decades witnessed both mechanistic advances, including the detailed mapping of homocysteine’s interference with endothelial nitric oxide and promotion of oxidative stress, and initial optimism about therapeutic B-vitamin supplementation. However, the lack of concrete outcomes in large-scale randomized trials ultimately prompted a critical re-evaluation of homocysteine’s role as a therapeutic target.

### 2.2. Mechanism of Cardiovascular Damage

A relationship between elevated homocysteine levels and cardiovascular disease (CVD) risk including coronary artery disease, cerebrovascular and peripheral artery disease has been documented [17,18].

The effects of severe hyperhomocysteinemia (HHcy) were studied in subjects (rats and humans) with homozygous deficiency in cystathionine-beta-synthase (CBS), an enzyme involved in homocysteine conversion to cysteine. In this cohort, there were higher levels of homocysteine up to 40-fold of the common levels. This condition was associated with a vascular event occurring in about half of this population, before the age of 30 [17].

Another genetic cause of HHcy is represented by the homozygous mutation of methylenetetrahydrofolate-reductase (MTHFR) [17,18].

Furthermore, according to recent evidence, the presence of particular single nucleotide polymorphisms associated with HHcy was correlated with an elevated risk of stroke [18].

A positive relationship between the number of coronary lesions and their complexity with homocysteine levels was observed. Indeed, HHcy determined the increased activity of HMG-COA reductase, resulting in increased cholesterol levels [17].

HHcy was positively related to increased systolic and diastolic blood pressure, mainly in women. Furthermore, induced HHcy in rats resulted in increased secretion of catecholamines that produced brain and cardiovascular systems damages [17].

HHcy is associated with the inflammation process, endothelial damage, and CVD. These effects are produced by different mechanisms such as increased vascular smooth muscle cell proliferation, increased collagen synthesis, and arterial wall elastic deterioration. Inflammation is mediated by NF-kB pathway activation in smooth muscle cells, resulting in increased CRP synthesis [17]. Homocysteine metabolism creates a series of intermediate molecules such as AdoHcy (S-adenosylhomocysteine), S-Hcy-proteins, Hcy-thiolactone, and N-Hcy-proteins. These substances are particularly elevated in CBS deficiency patients. Particularly, AdoHcy is associated with CVD and stroke. Indeed, according to several studies AdoHcy is considered a more severe indicator of CVD than homocysteine plasma levels. AdoHcy, in particular, has been shown to be a stronger predictor of cardiovascular risk than plasma homocysteine, as demonstrated in original studies and summarized in meta-analyses [19,20,21]. Furthermore, differently from Hcy, AdoHcy levels are not influenced by folate concentration. N-Hcy-proteins are responsible for pro-immunogenic, pro-amyloidogenic, pro-atherogenic, and pro-thrombogenic properties. Hcy metabolites are involved in endothelium damage, as studied in human umbilical vein endothelial cells (HUVECs). In these cell cultures, a dysregulation of the mTOR pathway (which plays an important role in vascular physiology) and autophagy was observed, resulting in endothelial dysfunction. With regards to Hcy thrombogenic properties, a recent study analyzed that urinary homocysteine (uHcy)-thiolactone correlated with fibrin clot lysis time (CLT) and elevated CLT was associated with worse prognosis. Furthermore, these metabolites cause a dysregulation of proteins involved in the coagulation pathway with the formation of N-Hcy-fibrinogen which has prothrombotic properties. Indeed, N-Hcy-fibrin clots, derived from N-Hcy-fibrinogen, are lysed slower by plasminogen because of its dysfunctional activation [22]. Thrombosis is also favored by higher levels of B-thromboglobulin and factor VIIc [17]. Plasma Hcy-thiolactone and total homocysteine (tHcy) levels are associated with development and progression of macrovasculopathy. This was demonstrated by a study that enrolled 120 Chinese patients with type 2 diabetes and 40 healthy controls. Furthermore, in diabetic patients with macrovasculopathy, plasma Hcy-thiolactone and tHcy levels were higher compared with patients without macrovasculopathy. Plasma Hcy-thiolactone levels were positively correlated with diabetes duration and negatively correlated with HDL. This can be explained by the activity of HDL-associated PON1 enzyme to eliminate Hcy-thiolactone [22].

HHcy also results in arterial stiffness, as a consequence of oxidative endothelial cell stress and reduced nitric oxide synthesis, associated with increased blood pressure in vitro and in animal studies [17].

## 3. What to Do: Evidence-Based Interventions

### 3.1. Focus on Folate, Vitamin B6, and B12

The B vitamins—particularly folate (vitamin B9), vitamin B6 (pyridoxine), and vitamin B12 (cobalamin)—play essential roles in metabolic homeostasis, DNA synthesis and repair, and homocysteine metabolism, the dysregulation of which is associated with increased cardiovascular and neurodegenerative risk [23,24]. These micronutrients are also involved in a wide range of physiological and pathological processes, particularly in older adults, individuals with metabolic syndrome, and those experiencing chronic inflammation or oxidative stress [25].

Recent guidelines from the European Society of Cardiology (ESC) and the American Heart Association (AHA) support the view that homocysteine should be regarded not as an isolated therapeutic target, but rather as a modifiable biomarker within a comprehensive cardiometabolic context [26].

These guidelines emphasize the importance of considering various biomarkers and risk factors in an integrated approach to cardiovascular risk management [26,27,28,29].

Genetic factors—most notably methylenetetrahydrofolate reductase (MTHFR) C677T polymorphism—can impair folate metabolism by reducing 5-methyltetrahydrofolate (5-MTHF) availability, thereby limiting homocysteine remethylation and increasing cardiovascular risk [30]. Furthermore, inter-individual differences in absorption and pharmacokinetics can significantly influence the metabolic efficacy of B vitamins in promoting homocysteine clearance [31].

**Figure 1:**
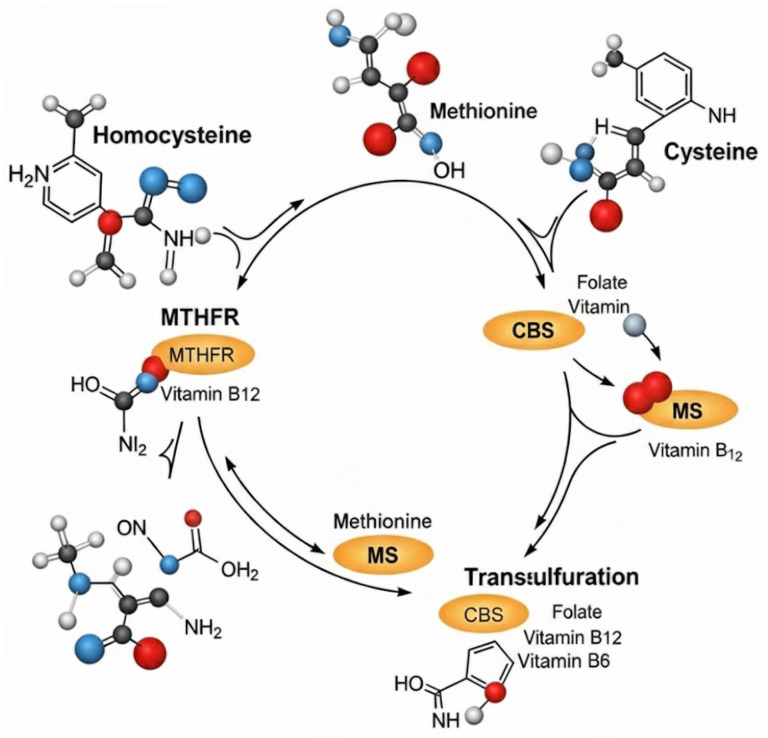
Homocysteine metabolism. (MTHFR: Methylenetetrahydrofolate reductase; CBS: cystathionine β-synthase; MS: methionine synthase).

The biological effects of B vitamins in homocysteine metabolism can be explained by their distinct yet interconnected biochemical pathways:

- Folate (vitamin B9): Folate functions in one-carbon metabolisms as a methyl donor via 5-MTHF, enabling the remethylation of homocysteine to methionine through the vitamin B12–dependent enzyme, methionine synthase [23]. Deficiency in folate impairs DNA synthesis and promotes hyperhomocysteinemia and megaloblastic anemia [32].
- Vitamin B6 (pyridoxine): Vitamin B6 is a coenzyme involved in amino acid transamination, neurotransmitter synthesis (e.g., serotonin, dopamine, GABA), and glycogenolysis. In homocysteine metabolism, B6 facilitates the transsulfuration pathway through the enzyme cystathionine β-synthase (CBS), converting homocysteine to cysteine [24].
- Vitamin B12 (cobalamin): Vitamin B12 is essential for methylation reactions and neurological integrity. As a coenzyme of methionine synthase and methylmalonyl-CoA mutase, it is critical for both homocysteine elimination and myelin maintenance. Deficiency can result from malabsorption (e.g., pernicious anemia, atrophic gastritis) and leads to hematologic and neurologic symptoms [32]. A schematic view of homocysteine metabolism is provided in Figure 1.

### 3.2. Supporting Scientific Evidence

Numerous studies and meta-analyses have assessed the impact of B-vitamin supplementation on homocysteine levels and cardiovascular outcomes. A 2013 Cochrane review confirmed that folic acid, B6, and B12 supplementation effectively lowers plasma homocysteine concentrations—a recognized surrogate marker of cardiovascular risk [33]. However, clinical trials have produced mixed results regarding cardiovascular endpoints: for example, the HOPE-2 study reported a modest but statistically significant reduction in stroke risk, whereas the NORVIT trial (Norwegian Study of Homocysteine-Lowering with B-Vitamins in Myocardial Infarction) found no cardiovascular benefit and even indicated potential harm in post-MI patients [8,34].

In neurology, the VITACOG study showed that B-vitamin supplementation significantly slowed brain atrophy in elderly subjects with mild cognitive impairment and elevated homocysteine, indicating a neuroprotective effect [35].

Observational studies also show associations between low plasma folate/B12 levels and an increased risk of cognitive decline and dementia, suggesting mechanisms related to impaired methylation, oxidative stress, and vascular dysfunction [23,25,30]. The bioavailability of B vitamins varies significantly based on their chemical form and route of administration. For instance, L-methylfolate bypasses the MTHFR-dependent activation step, offering greater efficacy in individuals with MTHFR polymorphisms. In malabsorption syndromes such as atrophic gastritis or celiac disease, sublingual or intramuscular B12 formulations may be required [30,36].

Therefore, understanding the pharmacokinetic profile and individual absorption status is essential for making informed clinical decisions.

In obstetric care, folic acid is universally recommended to prevent neural tube defects (NTDs), with optimal initiation at least one month before conception and continuation during the first trimester [37]

In conclusion, vitamins B6, B9, and B12 form an important triad for maintaining heart, nervous system, and metabolic health. Their role in regulating homocysteine is well documented, both mechanistically and biochemically. While supplementation effectively lowers plasma homocysteine levels, translating this into clinical benefit depends on multiple factors, including baseline vitamin status, MTHFR genotype, renal function, comorbid conditions, and lifestyle [38]. Recent studies suggest that the efficacy of supplementation can be improved with a personalized approach that takes into account each individual’s genetic profile. Individuals with the MTHFR C677T polymorphism may benefit more from supplementation with L-methylfolate (5-MTHF), which bypasses the enzymatic block and enhances homocysteine clearance [30,31]. Preliminary research in people with diabetes or moderate homocysteine levels has shown significant reductions in plasma levels when using formulations containing 5-MTHF, pyridoxal-5′-phosphate (the active form of vitamin B6), and methylcobalamin [36]. Moreover, co-supplementation with antioxidant micronutrients has shown potential for synergistic endothelial protection, though current evidence remains inconclusive [38]. The major take-home messages could be as follows:

- Focusing on homocysteine metabolism is crucial for developing strategies that protect cardiovascular and neurological health.
- Vitamins B6, B9 (folate), and B12 play complementary roles in the pathways of homocysteine remethylation and transsulfuration.
- While taking supplements can help lower homocysteine levels, the actual benefits depend on a mix of genetic, metabolic, and nutritional factors.
- Personalized strategies—like using active forms such as 5-MTHF, methylcobalamin, and pyridoxal-5′-phosphate—could boost effectiveness, especially for those who are genetically predisposed.
- In clinical practice, it is essential to think about formulation, bioavailability, and the method of administration.
- Further studies are warranted to identify optimal therapeutic targets and to delineate patient subgroups most likely to benefit from tailored supplementation strategies.

However, while biochemical correction of homocysteine levels is mechanistically sound, translating this into tangible cardiovascular benefits remains challenging. This necessitates a broader evaluation of modifiable lifestyle factors, as discussed in the following section.

### 3.3. Lifestyle Modifications and Risk Factor Management

Although elevated plasma homocysteine has historically been viewed as an independent cardiovascular risk factor, clinical trials focusing solely on its reduction through vitamin supplementation have mixed results in terms of clinical benefit. In particular, major randomized trials such as HOPE-2 and NORVIT have revealed that although supplementation of folic acid and B vitamins effectively reduces blood homocysteine, it does not lead to a significant decrease in the incidence of heart attacks, strokes, or cardiovascular deaths in high-risk populations [8,34]. These findings have shifted the attention of the scientific community from homocysteine as an isolated therapeutic target to a more integrated approach, in which homocysteine is viewed as part of a complex network of modifiable cardiovascular risk factors.

Lifestyle modifications—particularly in diet, physical activity, smoking cessation, and blood pressure management—have demonstrated synergistic benefits for cardiovascular health. Diets such as the Mediterranean diet and DASH, rich in folate, vitamins B6 and B12, and antioxidants, not only help maintain optimal homocysteine metabolism, but also improve endothelial function, reduce inflammation, and have a positive effect on lipid profiles [39,40].

Compared with supplements, whole foods offer a more sustained and bioavailable source of nutrients, along with additional benefits from polyphenols and dietary fibers. This has been confirmed by several cohort studies, including the well-known ATTICA study, which also revealed an interaction between diet and the MTHFR genotype [41] as well as additional analyses conducted in Greek populations [42].

In addition, an analysis of Dutch samples revealed that factors such as smoking, caffeine consumption, and physical activity have a significant impact on homocysteine levels [43].

Regular physical activity has been linked to moderate reductions in homocysteine levels, probably due to improved methylation capacity, increased renal clearance, and decreased systemic oxidative stress [44]. In addition, exercise improves blood vessel responsiveness and reduces blood pressure, two key factors that may mitigate the negative effects of elevated homocysteine. Guidelines recommend at least 150 min of moderate aerobic activity or 75 min of vigorous activity per week for significant benefits. Importantly, quitting smoking has been shown to lower plasma homocysteine levels, probably by reversing the oxidative and inflammatory burden caused by tobacco toxins, which alter homocysteine metabolism and deplete B-vitamin stores [45].

It is well established that hypertension, insulin resistance, and dyslipidemia—collectively referred to as metabolic syndrome—can exacerbate homocysteine-related vascular damage through synergistic mechanisms. These include endothelial dysfunction, the enhanced production of reactive oxygen species (ROS), and increased thrombotic risk. Consequently, focusing only on homocysteine reduction without addressing these concomitant conditions could prove insufficient or even clinically misleading. Indeed, expert recommendations emphasize that homocysteine should not be interpreted in isolation but within the broader context of cardiovascular risk factors [46].

A more comprehensive and multifactorial strategy that considers overall cardiovascular risk—including homocysteine, where appropriate—is likely to yield greater benefits in both primary and secondary prevention. In this context, physicians should avoid adopting a reductionist perspective that isolates homocysteine as a therapeutic target. Instead, interventions should aim to restore metabolic and vascular homeostasis through personalized lifestyle counseling, targeted nutritional therapy, and management of coexisting risk factors. Homocysteine should be regarded not as an isolated therapeutic target, but rather as a modifiable biomarker within a comprehensive cardiometabolic context.

### 3.4. Monitoring Homocysteine Levels

In recent years, measuring plasma homocysteine has received increasing attention, primarily due to its potential role as a modifiable risk factor for cardiovascular disease (CVD). Homocysteine is a sulfur-containing amino acid involved in methionine metabolism, particularly in the remethylation and transsulfuration pathways.

When these metabolic routes are disrupted, it can lead to HHcy, a condition associated with endothelial dysfunction, oxidative stress, and a prothrombotic state [47,48].

#### 3.4.1. When and Why Should It Be Measured?

Homocysteine testing is not recommended for routine screening in the general population, but it can be useful in well-defined clinical scenarios. Specifically, it may be indicated in the following:

Patients with premature cardiovascular or cerebrovascular events, especially in the absence of traditional risk factors [49];

Individuals with a history of recurrent venous thromboembolism or pregnancy-related complications such as recurrent miscarriage or preeclampsia; 

Young stroke patients with no clear underlying cause;

Cases of accelerated atherosclerosis or when investigating inherited metabolic disorders such as homocystinuria;

Unexplained cognitive decline, where elevated homocysteine levels may play a role in neurovascular impairment [8].

In clinical practice, we sometimes encounter young patients with unexplained ischemic events or women with recurrent pregnancy loss without a clear cause. In these contexts, homocysteine testing can be a useful piece of the diagnostic puzzle.

Although several studies have found an association between elevated homocysteine levels and cardiovascular risk, evidence from randomized controlled trials using B-vitamin supplementation to lower homocysteine has not consistently shown a reduction in cardiovascular events [34,50]. For this reason, testing should be targeted and clinically justified, rather than used routinely.

#### 3.4.2. Current Clinical Applications

Currently, homocysteine testing is used in specific, well-defined situations:

Screening for homocystinuria, especially in younger patients presenting with vascular events or ectopia lentis;

Nutritional assessment, particularly in cases of anemia, malabsorption, or following bariatric surgery, where deficiencies in folate or B vitamins may be suspected;

In patients at high cardiovascular risk, such as those with chronic kidney disease (CKD), where impaired renal clearance can lead to elevated homocysteine levels [51];

In obstetric settings, including preeclampsia, placental abruption, or recurrent pregnancy loss, especially when no other underlying cause is identified [52].

Current clinical guidelines do not recommend using homocysteine as part of routine cardiovascular risk assessment in the general population [53].

#### 3.4.3. At-Risk Populations

There are several groups in which elevated homocysteine levels are more frequently observed—often due to genetic predispositions, underlying medical conditions, or nutrient-related factors. In clinical practice, being aware of these populations can help tailor both diagnostic workups and treatment plans more effectively.

Genetic variants: The most studied genetic factor is MTHFR C677T polymorphism. Individuals who are homozygous (TT genotype) tend to have reduced enzyme activity, which affects the remethylation of homocysteine—especially when folate intake is insufficient [54].

It is not uncommon to find this mutation in patients with unexplained vascular events, particularly when traditional risk factors are absent.

Familial HHcy: A rare but important inherited disorder, often due to cystathionine β-synthase (CBS) deficiency, which can cause marked elevations in homocysteine levels. If undiagnosed, it significantly increases the risk of early thrombosis. In practice, this condition often emerges in younger patients with unusual vascular presentations.

Chronic kidney disease (CKD): In patients with moderate to advanced CKD, homocysteine tends to accumulate because of reduced renal clearance. Moderate hyperhomocysteinemia is almost universal in this population [8], and while it is not always a primary target for treatment, it can contribute to the already elevated cardiovascular risk.

Vitamin deficiencies: Deficiencies in folate, vitamin B6, and vitamin B12 interfere with the enzymatic pathways that keep homocysteine in check. These deficiencies can stem from various causes—poor diet, aging, malabsorption, or chronic medication use (e.g., metformin, methotrexate, proton pump inhibitors) [23].

In common experience, this is especially relevant in elderly patients or those in long-term polypharmacy, where subtle micronutrient imbalances are easily overlooked.

Why it matters: For these at-risk groups, testing homocysteine levels is not just an academic exercise. It can guide nutritional strategies, help identify inherited metabolic conditions, and in some cases, contribute to a more nuanced understanding of a patient’s cardiovascular risk profile.

## 4. What Not to Do: Misconceptions and Pitfalls

### 4.1. The Genetic Puzzle: MTHFR and Beyond

5,10-Methylenetetrahydrofolate reductase (MTHFR) is an essential enzyme in folate and homocysteine (Hcy) metabolism [55]. This enzyme catalyzes the NADPH-linked reduction in 5,10-methylenetetrahydrofolate (5,10-methylene-THF) to 5-methyltetrahydrofolate (5-methyl-THF). The last molecule is a methyl group donor for the conversion of homocysteine to methionine (Met) in the reaction catalyzed by methionine synthase (MS) [56]. Vitamin B_12_ acts as a cofactor during this process. Met is then converted to S-adenosylmethionine (SAM), which is a crucial methyl group donor for various reactions in the body, including the methylation of DNA [57]. In addition, Hcy can be converted to cysteine through a transsulphuration process involving the enzyme Cystathionine-β-synthase (CBS) and vitamin B_6_. Under Met deficiency conditions, CBS is not activated and MTHFR is not inhibited by SAM. As a result, Hcy is converted back into Met, while cysteine contributes to glutathione synthesis or is degraded to taurine [23]. The substrate 5,10-methylenetetrahydrofolate is used by thymidylate synthase to methylate dUMP to dTMP, which is the only required source of thymidine for DNA synthesis and repair [58].

MTHFR enzyme activity is important for homeostasis of the serum homocysteine level [59]. 

The reduced activity of the MTHFR pathway inhibits the production of 5-methyltetrahydrofolate and can lead to accumulation of the substrate. Decreased levels of the MTHFR product cause increased levels of homocysteine, decreased levels of blood folate and displacement means for folate synthesis and DNA repair [58]. Variations in the *MTHFR* gene can lead to disruptions in multiple physiological and biochemical reactions in the body, such as modifications in the methylation of nucleic acids and proteins and the regulation of the cell cycle [60].

The *MTHFR* gene is located at the end of the short arm of chromosome 1 (1p36.3), and the DNA sequence for this gene is approximately 2.2 kilobases (kb) and includes 11 exons [61,62].

Genetic variants in *MTFHR* may result in decreased enzyme activity, leading to alterations in homocysteine levels [63].

However, it is not clear whether common variants are a major risk factor for diseases because their association with health conditions varies among different populations [64,65].

Currently, hundreds of single nucleotide polymorphisms (SNPs) have been identified in the *MTHFR* gene [60]. C677T and A1298C represent the two polymorphisms in *MTHFR* which are most frequently investigated in clinical practice [63]. In the general population, 60–70% of individuals will have at least one of these variants, 8.5% will be homozygous for 677C > T or 1298A > C, and 2.25% will be compound heterozygous. Overall, 10% of the population will be homozygous or compound heterozygous for these two polymorphisms [66,67].

C677T polymorphism involves the substitution of cytosine with thymine at position 677, resulting in an amino acid change from alanine to valine in the enzyme [16,55,60].

This site is crucial in terms of the binding of flavin adenine dinucleotides (FADs) and enzyme stability. The 677T allele encodes a thermolabile enzyme with reduced activity and less affinity for its cofactor, FAD. Each copy of the 677T allele results in 35% of reduced enzyme activity [56]. The 677TT homozygotes are believed to have reduced levels of active folate (5-methyl-THF) and increased plasma levels of Hcy because it cannot be remethylated to Met [16]. In individuals who are heterozygous for the *MTHFR* C677T mutation, enzyme function is reduced to 65% of normal function. In people who are homozygous for *MTHFR* C677T, there is only 30% of normal enzyme function [68]. The 677TT homozygotes are believed to have reduced levels of active folate (5-methyl-THF) and increased plasma levels of Hcy because it cannot be remethylated to Met [16]. In individuals carrying the C677T variant, enzyme activity is decreased to approximately 67% and 25% for those with one copy (heterozygous) and two copies (homozygous) of the T allele (*MTHFR* C677T polymorphism), respectively [56].

Hyperhomocysteinemia, induced by this genetic variation, is considered an independent risk factor for cardiovascular diseases (CVDs), stroke, and venous thrombosis, which triggers the proliferation of vascular smooth muscle and endothelial injury and contributes to hypertension [69].

The 677T allele has been associated with an increased risk of overweight and obesity, as well as associated diseases, which include dyslipidemia or insulin resistance [56].

Another commonly encountered polymorphism occurs at position 1298 on the *MTHFR* gene and involves the substitution of adenine with cytosine, resulting in an amino acid change from glutamate to alanine in the enzyme. This mutation also produces reduced enzyme activity, although not to the same extent as that caused by C677T polymorphism [70].

*MTHFR* genetic testing investigates two common variants, 677C > T and 1298A > C, and is frequently ordered by several providers due to the association of those variants with several multifactorial diseases, including cancer, autism, mental disorders, cardiovascular diseases, and congenital malformations.

The relationship between polymorphisms (i.e., common variants) and the risk for multifactorial diseases is complex and influenced by numerous aspects, including ancestry [63].

Genetic testing of the *MTHFR* gene may be used to confirm the diagnosis of an inherited hyperhomocysteinemia caused by MTHFR deficiency [71].

However, a 2013 Practice Guideline from the American College of Medical Genetics and Genomics (ACMG) states that there is growing evidence that *MTHFR* polymorphism testing has minimal clinical utility and, therefore should not be ordered as a part of a routine evaluation [72]. The American Heart Association (AHA) does not recommend MTHFR mutation testing in routine clinical practice in any patient group [68].

As 677C > T and 1298A > C polymorphisms occur at high rates in the general population, and there are no clinically significant interventions that could be offered to carriers of the variants in heterozygous, homozygous, or compound heterozygous states, it is not useful to offer genetic testing for these variants [73].

These variants should be considered part of an additive polygenic model of genes and environment, rather than high penetrant variants because the relationship between *MTHFR* polymorphisms and homocysteine levels or risk for diseases may depend on the presence of other genetic variants in the same individual. If the goal is to correct elevated homocysteine levels through supplementation, it may be more reasonable to test homocysteine levels directly before performing a genetic test for *MTHFR* [63].

### 4.2. Avoiding Unnecessary Supplementation 

The enzymes involved in the homocysteine transsulfuration pathway and the remethylation cycle rely on B vitamins, and taking into account for the specific gene status, supplementing with high dose of B vitamins (B6, B9, B12) leads to substantial decreases in homocysteine plasma levels, with a reduction of 27–32% observed in controlled trials [74,75]. For this reason, although a well-balanced diet caters for most nutritional requirements, self-prescribing B vitamins (B6, B9 and B12) is becoming a common preventive approach to trying to reduce hyperhomocysteinemia, a condition linked to increased cardiovascular and neurological risk [75,76,77,78]. But unnecessary supplementation and the self-prescription of vitamins, dietary supplements, or medications represent a public health problem due to risk of dangerous side effects, interactions between drugs, and disease masking [79].

When supplemented in small doses, many vitamins are safe, but long-term consumption of mega-doses may cause harm. There is some evidence that the arbitrary and uncontrolled use of vitamin preparations might be harmful. For example, amongst 38,772 older women (mean age, 61.6 years) who participated in the Iowa Women’s Health Study users of multivitamins, B6, B9, or minerals had an increased risk of mortality when compared with nonusers. This study points out that the self-initiated, uncontrolled intake of vitamin or mineral supplements in elderly women could be harmful. [80,81]. Vitamins and dietary supplements are frequently self-administered by older individuals to relieve symptoms of their current illnesses [82]. Physiological changes in older adults, such as altered metabolism and decreased renal function, make them more susceptible to vitamin toxicity and adverse reactions. Self-prescribing bypasses professional guidance, increasing the likelihood of dosing errors and unrecognized interactions or overdoses [83]. Vitamin B overdose can lead to a range of clinical consequences, which vary depending on the specific B vitamin involved. The most well-documented risks are neurological symptoms, particularly from vitamin B6, and a range of acute symptoms from excessive vitamin B12. High-dose folic acid can also mask B12 deficiency, worsening neurological damage.

High doses of vitamin B6 can cause sensory and motor neuropathy, leading to symptoms such as numbness, tingling, and muscle weakness. This risk is especially notable in populations with high supplement use, such as bariatric surgery patients [84,85,86]. Pyridoxine toxicity is linked to injury of sensory neurons, possibly through disruption of neurotransmitter (GABA) signaling in peripheral nerves [84]. Excessive vitamin B12 intake has been associated with acne, palpitations, anxiety, restlessness (akathisia), facial redness, headache, and insomnia. These symptoms typically resolve after stopping supplementation and do not usually cause lasting harm [87].

Serious or permanent complications from B12 overdose are rare, but such cases highlight that even water-soluble vitamins can cause adverse effects at high doses [87]. High doses of folic acid can mask the blood-related symptoms of vitamin B12 deficiency, allowing neurological damage to progress undetected and potentially worsen cognitive and nerve problems. This is particularly concerning in older adults and those with conditions affecting B12 absorption [12].

Excessive doses of vitamin B can influence the activity of cytochrome P450 enzymes, which are crucial for drug metabolism. High concentrations of certain B vitamins, especially riboflavin (B2), can inhibit the catalytic activity of cytochrome P450 3A4, potentially altering the metabolism of drugs processed by this enzyme. High doses of vitamin B group, particularly riboflavin, have been shown to inhibit the hydroxylation of drugs like diclofenac by cytochrome P450 3A4. This inhibition can lead to higher blood concentrations of the drug, as its breakdown is slowed. The inhibitory effect increases with higher doses of vitamin B, as seen by a significant rise in drug levels when more vitamin B is co-administered [88]. In conclusion, B-vitamin supplementation is a proven, effective strategy to lower homocysteine in people with elevated levels or B-vitamin deficiencies. However, it is best to confirm deficiency or elevated homocysteine plasma levels, to take into account the specific gene status before self-prescribing and to monitor levels during supplementation to avoid the risk of dangerous side effects and interactions between drugs. Professional guidance ensures appropriate dosing, reduces risks, and helps avoid severe health consequences.

### 4.3. Interpreting the Evidence: A Word of Caution

Folic acid and cyanocobalamin deficiencies are common conditions in older adults. This could be explained by an increased prevalence of atrophic gastritis with achlorhydria, alcohol consumption, and drug use (methotrexate, metformin, and niacin). Many pathologic conditions can also cause these deficiencies like diabetes mellitus type 2, chronic renal insufficiency, and hypothyroidism. According to the Framingham study, low concentrations of one or more B vitamins were associated with HHcy. The correction of these deficiencies, through B-vitamin administration, proved to reduce homocysteine levels but with controversial effects on cardiovascular outcomes. Indeed homocysteine metabolites, as plasma AdoHcy and Hcy-thiolactone, are not influenced by B vitamins supplementation, and for this reason they are considered as Hcy-independent CVD risk factors [22].

According to the VITATOPS trial (VITAmins TO Prevent Stroke), daily supplementation of folic acid, cyanocobalamin, and pyridoxine in patients with a recent history of transient ischemic attack or stroke, did not influence markers of vascular inflammation, endothelial dysfunction, and hypercoagulability [89].

The NORVIT trial enrolled patients with established CVD divided into two groups: one receiving B vitamins and the other one placebo. It demonstrated that the reduction in homocysteine levels was not associated with decreased incidences of MI and stroke [34]. 

The HOPE-2 (Heart Outcomes Prevention Evaluation Study-2) proved that supplementation with B vitamins did not reduce the mortality for CVD, MI, or stroke (with only a reduction of 25% in stroke incidence risk) and instead caused a larger number of hospitalizations for unstable angina [8,90]. Stroke risk reduction is also underlined by a recent metanalysis that evaluated B vitamins supplementation [91]. For this reason, homocysteine has been defined “an innocent bystander” in the development of CVD [90]. In contrast to these observations, another trial documented a decreased carotid intima–media thickness and a decreased incidence of complications after percutaneous coronary intervention, following B-vitamin complex administration.

There are many hypotheses about the inefficacy of B-vitamin supplementation such as insufficient doses administrated or the short duration of interventional trials. Whereas for trials which enrolled patients in secondary prevention on therapy with aspirin, statins, and other drugs, the benefits derived from B-vitamin supplementation may be hidden by using these drugs [92].

A summary overview of these studies is provided in Table 1. In Table 2 is provided a schematic correlation between homocysteine, vitamin supplementation and cardiovascular risk.

For this reason, current guidelines, such as the American Heart Association (AHA), do not recommend neither B-vitamin supplementation nor routine screening for elevated homocysteine levels [53].

## 5. Therapeutic Approaches to Hyperhomocysteinemia: Current Paradigms and Future Directions

Despite a clear biochemical efficacy of B-vitamin supplementation in reducing plasma homocysteine levels, the therapeutic management of hyperhomocysteinemia in the context of cardiovascular disease (CVD) remains nuanced and largely directed towards specific patient populations, rather than widespread primary prevention in individuals with mild-to-moderate elevations.

However, targeted intervention remains critical in specific scenarios. For individuals with severe hyperhomocysteinemia (e.g., >100 µmol/L), often linked to genetic disorders like classical homocystinuria (cystathionine beta-synthase deficiency) or severe MTHFR deficiency, aggressive therapy is mandated to prevent devastating neurological, ocular, and thrombotic complications. Treatment typically involves high doses of folic acid (0.4 mg to 5 mg daily), vitamin B12 (0.5 mg to 1 mg daily), and vitamin B6 (10 mg to 50 mg daily), tailored to patient responsiveness [93]. In MTHFR polymorphisms, particularly the C677T variant, supplementation with L-methylfolate (0.8 mg to 1.0 mg daily) is often favored over synthetic folic acid due to its superior bioavailability and bypass of the impaired MTHFR enzyme. Additionally, betaine (trimethylglycine—TMG), typically in doses of 4 g/day or more, is a cornerstone therapy for certain types of homocystinuria and MTHFR deficiencies, acting as an alternative methyl donor. For cases of elevated homocysteine driven by confirmed B-vitamin deficiencies, supplementation is appropriate to correct the underlying nutritional deficit. Future pharmacological strategies may focus on personalized approaches, considering genetic predispositions and individual metabolic profiles, to identify subsets of patients who might derive a clinical benefit from homocysteine-lowering therapies beyond the correction of overt deficiencies.

## 6. Conclusions

In light of the cumulative evidence, it is increasingly recognized that homocysteine functions more reliably as a marker of cardiovascular risk rather than a modifiable therapeutic target in patients with established vascular disease. As early as 2002, Wald et al. [2]. emphasized that although elevated homocysteine was associated with increased cardiovascular risk, lowering homocysteine in patients with advanced atherosclerosis may be ineffective, as the disease may already be beyond biochemical repair Subsequent mechanistic insights provided by Ganguly and Alam [17] further support this view, highlighting how chronic elevations in homocysteine contribute to oxidative stress, endothelial dysfunction, and vascular inflammation—processes that, once entrenched, may not respond adequately to late-stage vitamin intervention. In this context, homocysteine may be considered a biological signature of prior vascular injury rather than an actionable risk factor when detected too late. This paradigm has led to growing advocacy for earlier screening and intervention, particularly in populations with genetic predispositions (e.g., MTHFR polymorphisms), renal dysfunction, or low dietary folate intake.). Despite compelling epidemiological and genetic evidence linking elevated homocysteine levels to an increased risk of cardiovascular disease (CVD) and stroke, a significant discordance persists between observational findings and the outcomes of randomized controlled trials (RCTs) of B-vitamin supplementation. Landmark intervention trials like HOPE-2 (2006) [8], NORVIT (2006) [34], and VITATOPS (2010) [89], while effectively lowering plasma homocysteine concentrations, largely failed to demonstrate significant reductions in “hard” cardiovascular endpoints such as myocardial infarction, stroke (with only modest effects observed in some subgroup analyses), or all-cause mortality. Importantly, these pivotal trials predominantly involved elderly patients (typically >60 years of age) with pre-existing cardiovascular or cerebrovascular disease, highlighting that intervention occurred at an advanced stage of vascular pathology. This consistent lack of clinical benefit in the context of effective biochemical reduction, particularly in such a vulnerable and already diseased population, has led to the widely accepted “too late” hypothesis. In fact, conflictual data are in two meta-analyses. In 2011, ref. [94]. Zhoy concluded that folic acid supplementation had no significant effect on major cardiovascular events, myocardial infarction, or all-cause mortality. They also showed no significant effect on stroke (Relative Risk, 0.89; 95% CI, 0.78–1.01) but in more recent meta-analyses have provided slightly different interpretations, especially concerning stroke. the meta-analysis by Li Y [95] found a 10% lower risk of stroke (RR 0.90; 95% CI 0.84–0.96; *p* = 0.002) with folic acid supplementation, a 4% lower risk of overall CVD (RR 0.96; 95% CI 0.92–0.99; *p* = 0.02), and no significant effect on coronary heart disease. Crucially, this study highlighted that the benefit for both stroke and overall CVD was more pronounced among participants with lower plasma folate levels at baseline and without pre-existing CVD, and in studies with larger reductions in homocysteine levels. This concept posits that once atherosclerotic damage and vascular pathology are well established, simply modulating a single risk biomarker like homocysteine may be insufficient to alter the disease trajectory. This underscores the need for a nuanced understanding of homocysteine’s role, differentiating its potential as a causal factor in early disease development from its limited utility as a therapeutic target in established vascular disease, thereby guiding future precision medicine approaches in cardiovascular prevention. Taken together, these data reinforce the need to reframe homocysteine assessment as a tool for early risk stratification, ideally in midlife or before the onset of overt vascular disease, rather than as a late-stage therapeutic target. In summary, it is crucial to choose the right timing for homocysteine measurement—early assessment may be key to effective intervention, while late testing risks missing the window for meaningful clinical benefit.

## Figures and Tables

**Table 1:** Randomized comparison study on homocysteine.

Trial	Population	N (Treatment)	Age	Condition at Baseline	Follow-Up	Hcy Reduction
HOPE-2	>55 Y established CVD or diabetes	2958	69 Y	83% CVD; 40% diabetes; chronic stage	5 y	25%
NORVIT	Post-MI within 7 days	937	63–64	Acute MI survivors; optimized therapy	3.5 y	27%
VITATOPS	Recent stroke or TIA (≤7 mo)	8164	62 ± 12	Secondary prevention in cerebrovascular desease	3.4 y	similar

**Table 2:** Summary: Vitamin, homocysteine, and cardiovascular risk.

**Common Causes of** **B Vitamins Deficiency**	Atrophic gastritis (achlorhydria), alcohol use, medications (metformin, methotrexate, niacin), chronic conditions (T2DM, CKD, hypothyroidism).
**Mechanism of Action**	B vitamins (folic acid, B12, B6) lower plasma homocysteine (Hcy), a known marker of cardiovascular risk.
**Framingham Study**	Linked low B vitamin levels to hyperhomocysteinemia (HHcy), which correlates with increased CVD risk.
**Positive Findings**	↓ Stroke risk in meta-analysis ↓ Carotid intima–media thickness (one trial) Association between HHcy and subclinical atherosclerosis [92]
**Negative Findings (RCTs)**	VITATOPS: No benefit in stroke/TIA prevention CHAOS-2: No effect on CAD events NORVIT: No MI/stroke reduction HOPE-2: No effect on CVD death; ↑ unstable angina hospitalizations
**Potential Explanations**	Inadequate dosing or trial duration Confounding from standard secondary prevention therapy (aspirin, statins, etc.) [92]
**Current Guidelines (AHA)**	Do not recommend routine homocysteine screening or B-vitamin supplementation for cardiovascular prevention [53].
**Alternative Hypothesis**	Homocysteine may be an “innocent bystander” rather than a causative agent in CVD [90].
**Independent Risk Factors**	AdoHcy and Hcy-thiolactone not affected by B-vitamin supplementation; may contribute to CVD independently [22].

## Data Availability

The data from this manuscript are derived from publicly available published clinical trial and study results.
